# Cheap Genes and Academic Conflict

**DOI:** 10.1100/tsw.2001.11

**Published:** 2001-03-16

## Abstract

A story about a German research group that supplies free genes leads off the news in Nature this week. Science leads with a story about academics who are offended by a recent suggestion by the US government that they disclose ‘institutional conflicts of interest’ in scientific research.

Hot off the *Nature* presses: You have seen the map, now get the gene - free! A German team, led by Stefan Wiemann of the German Cancer Research Centre in Heidelberg, is now offering more than 1500 synthetic human genes free of charge to academic and industrial scientists, reports *Nature* in its lead story. The synthetic genes are called complementary DNA, or cDNA, and they can be used to synthesize the same protein as encoded by the real gene, reports *Nature*. Putting the artificial cDNA in a freely available library and allowing any researcher to check one out whenever needed should speed up the study of genetic disease. It is such a good idea that several other groups are doing it, too, says *Nature*. The Kazusa cDNA project in Japan pioneered the systematic production of cDNA in the mid-1990s and the National Institutes of Health are also jumping on the bandwagon. With all those scientists hard at work, and now that the human genome map has shown that there are only 32,000 genes in a strand of human DNA, the goal of a complete cDNA library seems within reach, reports *Nature*. And scientists will get there even faster if they avoid duplicate work. So they did what any technically savvy scientist would do in that situation: they started a web site. Science did not cover this story.

Following the tragic death of a young man in a gene therapy experiment conducted at the University of Pennsylvania, and the subsequent disclosure of the university's financial stake in the company that hoped to profit from the research, former Secretary of Health and Human Services (HHS) Donna Shalala led an effort to establish clear guidelines for conducting profitable research at universities, reports *Science* in their lead story. The HHS finally issued draft guidance last week suggesting that researchers' potential conflicts be disclosed to Institutional Review Boards and patients. Academics blew a gasket. According to *Science*, four major education organizations and the Federation of American Societies for Experimental Biology, wrote a letter asking that the HHS guidelines be withdrawn. Greg Koski, director of the Office for Human Research Protections(OHRP), was "taken aback" by the "misleading" response from academia, reports *Science*. It's misleading because as Koski told *Science*, "We haven't issued any guidance yet." The statement was a draft, not an official guidance, said Koski, "and you can't withdraw something that hasn't been issued." *Nature* did not cover this story.

Claiming "the genie is out of the bottle," an Italian team announced plans to clone a human, report both *Science* and *Nature*. The team claims to have an unnamed-but-unlimited source of funding, a top-notch anonymous team of scientists, and a lab in an undisclosed country. Scientists universally condemned the mysterious plan.
